# Sinonasal glomangiopericytoma treated with preoperative embolisation and endoscopic sinus surgery

**DOI:** 10.3332/ecancer.2016.692

**Published:** 2016-11-14

**Authors:** Elizabeth Psoma, Petros D Karkos, Stamatia Dova, Michail Gavriilidis, Konstantinos Markou, Constantinos Kouskouras, Afroditi Haritanti, Stefanos Finitsis

**Affiliations:** 1 Department of Radiology and Interventional Radiology, AHEPA Hospital, Aristotle University of Thessaloniki, Thessaloniki 54621, Greece; 2 Department of Otolaryngology-Head and Neck Surgery, AHEPA Hospital, Aristotle University of Thessaloniki, Thessaloniki 54621, Greece

**Keywords:** sinus, nose, glomangiopericytoma, endoscopic sinus surgery, embolization

## Abstract

Sinonasal glomangiopericytoma is a benign rare tumour of pericytes that accounts for less than 0.5% of all sinonasal tumours. It is an indolent tumour with a macroscopic appearance of common inflammatory polyps.

We report the case of a 55-year-old male who presented with right nasal obstruction. CT and MRI examinations demonstrated a soft-tissue mass that obstructed mainly the right nasal cavity. Biopsy revealed glomangiopericytoma. The tumour was treated with preoperative embolisation followed by complete endoscopic resection. Very few cases have been reported to be treated in this way.

## Introduction

Sinonasal glomangiopericytoma (GPC) is a rare mesenchymal tumour of the sinonasal tract characterised by a prominent perivascular growth pattern with a low malignant potential [1–4]. This entity was first described as sinonasal haemangiopericytoma but was found clinicopathologically distinct from the conventional haemangiopericytoma that generally arises in soft parts [1–4]. WHO’s classification synonyms are ‘glomangiopericytoma’ and ‘myopericytoma’ because of its similarity with glomus tumours [5]. This tumour usually presents with nasal obstruction or repeated episodes of epistaxis [6, 4]. Endoscopic surgery is preferred following preoperative embolisation because of high vascularity that these lesions often exhibit.

We report the case of a 55-year-old male who was treated with preoperative embolisation with microparticles and complete endoscopic excision. We describe the clinicopathologic, radiologic, and surgical characteristics of the tumour and review the literature.

## Case report

A 55-year-old male presented to the ENT outpatient clinic complaining of right nasal blockage during the last six months. Endoscopy revealed a polypoid mass that filled the right nasal cavity and caused deviation of the lateral nasal wall. The patient’s medical history and laboratory studies were unremarkable.

**Figure 1.**
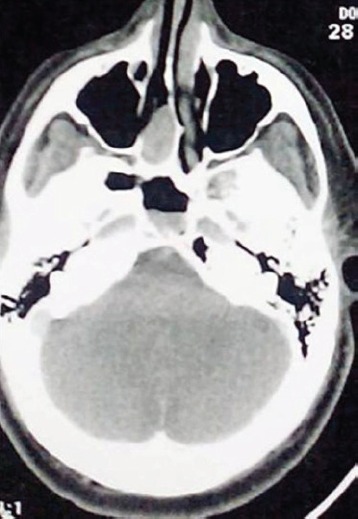
CT showing a soft tissue mass from the right nasal cavity extending to the posterior ethmoid air cells. Inflammatory changes are present to the right sphenoid sinus.

A computed tomography (CT) scan showed a well-defined, sharply enhancing mass lesion in the right nasal cavity extending into the posterior ethmoid air cells with minimum projection to the nasopharynx. The medial wall of the orbit was preserved, and there was no intracranial extension. The mass measured 4 x 3 x 1.5 cm. Retained secretions and inflammatory changes to the sphenoid and maxillary sinuses were also seen. No lymphadenopathy was identified (Figure 1).

**Figure 2.**
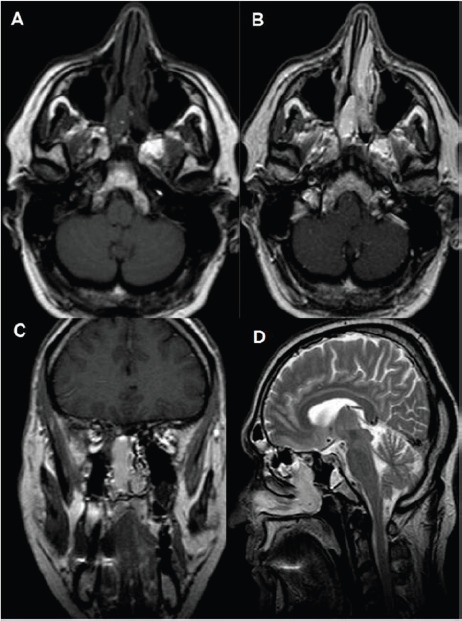
MRI (a) T1WI showing mainly hypointense mass in the right nasal cavity and posterior ethmoid air cells with retained secretions and inflammatory changes to the left ethmoid air cells (b) T1WI GD axial and (c) Coronal images showing the expansive soft tissue mass well-defined with intense enhancement (d) T2WI sagittal plane showing high signal intensity of the lesion.

Magnetic Resonance Imaging (MRI) revealed an ovoid encapsulated mass occupying the right nasal cavity extending into the ethmoid air cells and nasopharynx. Enhancement of the mass was heterogeneous on T1-weighted images following intravenous administration of gadolinium. The signal was heterogeneous on T2-weighted images (Figure 2a-d).

**Figure 3.**
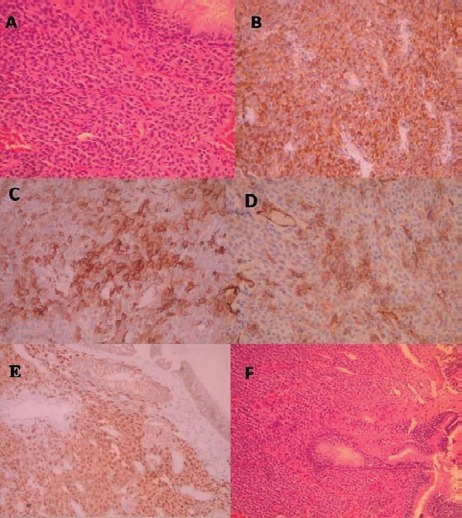
(a) Tumour of the nasal mucosa with moderate cellularity composed of ovoid to spindle cells with ovoid bland nuclei (H+E X 100) (b) Tumour cells are positive for vimentin (Immunostain X 200) (c) Tumour cells are focally positive for SMA (Immunostain X 200) (d) Few tumour cells are positive for CD34 (Immunostain X 200) (e) tumour cells show moderate to strong nuclear positivity for β-Catenin (ImmunostainX200) (f) Tumour cells of the nasal mucosa (H+E).

A biopsy was performed after preparation for any possible bleeding including nasal vasoconstrictors and general hypotensive anaesthesia, and this showed glomangiopericytoma. Histological examination of the biopsy specimen showed sheets of benign spindled cells arranged in a syncytium separated by prominent vessels. Pleomorphism, necrosis, and increased mitoses were not present. Immunohistochemistry showed positivity to vimentin, SMA, with few cells positive with CD34 and negative reactions with S-100 protein, desmin, and myogenin (Figure 3a-f).

**Figure 4.**
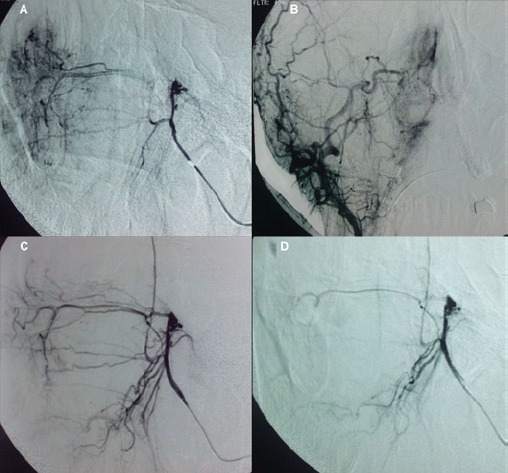
DSA (a, b) angiography showing branches of the right sphenopalatine artery (c, d) angiography after embolisation showed complete devascularisation of the tumour.

Because of the bleeding propensity of the tumour, preoperative embolisation was planned. Under general anesthesia and systemic heparinisation, the right common femoral artery was punctured and a 5F diagnostic catheter was used to selectively catheterise both external carotid arteries. The angiographic control demonstrated arterial supply to the mass by both internal maxillary arteries (Figure 4a, b). Although the tumour blush was mild, it was decided to perform the embolisation with the aim of diminishing perioperative blood loss and facilitating complete removal.

**Figure 5.**
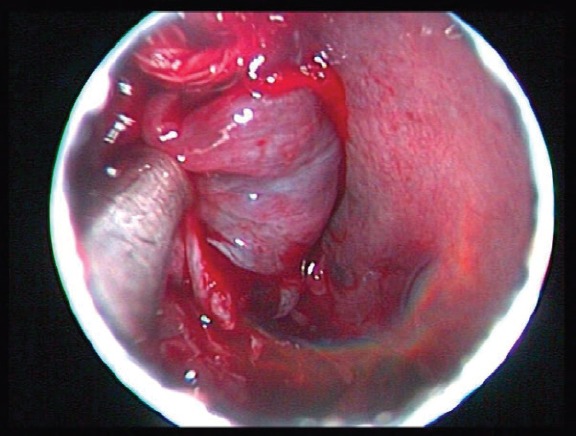
Intraoperative photograph showing the lesion arising from the middle turbinate.

With a coaxial technique, both internal maxillary arteries were sequentially catheterised with a microcatheter (Progreat, Terumo, and Tokyo, Japan). The vessels were embolised with microparticles measuring 350–500 *μ*m in size (Embospheres, Boston Scientific, Massachusetts, USA ). The final angiographic control showed complete devascularisation of the tumour (Figure 4c, d). Immediately after embolisation, the patient was transferred to the operating theatre. The patient underwent an endoscopic right middle meatal antrostomy followed by a ‘powered’ reduction of middle turbinate using microdebrider, anterior’, and posterior ethmoidectomy and a sphenoidotomy. The axilla of middle turbinate was left intact to guide as landmark for future surgery. The lesion arose from the middle turbinate and extended to the anterior wall of the sphenoid sinus (Figure 5). Complete resection of the tumour mass was achieved. Histological examination of the surgical specimen confirmed the former biopsy. The postoperative course was uneventful, and the patient was discharged a week later. The patient is under regular follow-up, and one year later he remains disease-free.

## Discussion

Sinonasal glomangiopericytoma (GPC), also known as haemangiopericytoma (HPC), was first reported by Stout and Murray in 1942 [1].

GPC is a rare, mesenchymal tumour of the sinonasal tract characterised by a prominent perivascular growth pattern with a low malignant potential, accounting for less than 0.5 % of sinonasal tumours. The proposed cell of origin is a modified perivascular glomus-like myoid cell [2, 3, 5].

The World Health Organisation in 2005 identified it as a unique sinonasal low malignancy lesion and proposed the term ‘glomangiopericytoma’ and ‘myopericytoma’ because of its similarity with glomus tumours.

This tumour has been known as HPC-like tumour or sinonasal HPC [5, 6].

GPC belongs to the category of borderline and low-malignant-potential soft tissue tumours of the nose and paranasal sinuses. It differs from conventional soft tissue HPC in location, histologic findings. and biologic behaviour with lower rates of metastasis and mortality as compared to soft-tissue HPC [5–7]. The presence of strong nuclear ß-catenin expression supports a mutation in the *CTNNB1* gene [8].

Patients across a broad age range may be affected, predominantly in the sixth and seventh decade of life in both genders, with a small predilection in females. Clinically, it may present with nasal obstruction or repeated episodes of epistaxis. It only rarely may provoke pain because of local infiltration, headache, vision impairment, and local swelling [5, 7]. Several etiological factors have been proposed like trauma, corticosteroid use, hypertension, and pregnancy but are not widely accepted [7, 9].

Usually because of the symptoms, patients are first evaluated endoscopically. Exact tumour extension is better appreciated with CT and MRI. Computed tomography invariably shows a soft-tissue mass with strong enhancement after contrast administration that may be mistaken for an inflammatory polyp. On T1-weighted MR images, the mass appears solid isointense with strong contrast enhancement, while on T2-weighted images the signal varies from iso- to hypo-intense, differentiating it from high-intensity inflammatory fluid. The vessel supply to the GPC is best displayed to digital subtraction angiography and is used to plan a preoperative embolisation. Transarterial embolisation is indicated in order to prevent massive surgical haemorrhage [10].

Sinonasal GPC is an indolent tumour with excellent prognosis after surgical resection. Most specimens are fragmented during endoscopic removal so the gross appearance of these lesions is generally not helpful. Watanabe *et al* [11] recognised at least three histologic subtypes: 1) tumours that are identical to soft tissue HPC and exhibit aggressive, locally destructive behaviour, and distal metastases 2) GPC (true HPC) where cells exhibit various degrees of myoid differentiation mimicking normal pericytes and no metastatic potential, and 3) tumours intimately related to peripheral glomus tumours.

Differential diagnosis includes vascular and other tumours such as solitary fibrous tumour, lobular capillary haemangioma (pyogenic granuloma), angiofibroma, and glomus tumour. Solitary fibrous tumours (SFTs) consist of a cellular spindle cell proliferation effacing submucosal structures with staghorn-type vessels. They are rare in the sinonasal tract. Angiofibromas have abundant stromal collagen and a prominent vascular stroma. Lobular capillary haemangiomas (LCHs) in contrast to the GPC have a lobular architecture with staghorn-type vessels in the interlobular stroma [12]. Glomus tumour does not have staghorn-like vessel proliferation, and it is usually localised in distal extremities [13].

The therapy of choice is definitely wide field complete excision. Endoscopic surgery is preferred, because it offers more advantages compared with the external surgery. The overview is better and can establish more precisely the tumour insertion and its extension. Furthermore, loss of blood is decreased, the risk of damaging the lacrimal structures is reduced, and the natural physiology of the nose can be maintained. Factors not favourable for endoscopic surgery are septal deviation, tumours which are large or highly vascular and have orbital extension. Nevertheless, intracranial extension and pterygopalatine fossa involvement are not limits for endoscopic approaches [14, 15].

When the tumour exhibits high vascularity, endoscopic resection is not excluded provided preoperative embolisation is performed. The benefits of this method are intraoperative bleeding reduction with better inspection of the tumour bed.

We believe that preoperative embolisation prior to endoscopic excision to be the treatment of choice of large or highly vascularised tumour in order to lower the risk of the procedure. In the literature, preoperative embolisation before resection of GPC has been reported in less than 23 patients [14, 16].

Local recurrence has been reported in 7–20% of cases, mainly because of incomplete surgical resection. Metastatic rates (5–10%) of these tumours are generally lower than those (12–60%) of HPC at other sites. Radiotherapy and chemotherapy can be used as both adjuvant therapy and palliation therapy for metastatic disease [14, 16].

## Conclusion

Sinonasal GPC has a relatively indolent behaviour with a potential for local recurrence but not metastatic disease. The diagnostic algorithm involves endoscopy, CT and MRI for lesion characterisation and definition of lesion extent. Angiography with superselective embolisation is indicated especially in the case of large or highly vascular tumours. It helps to reduce preoperative blood loss and operation time and adds to the safety of the procedure. Complete surgical resection is the mainstay of treatment and may be performed endoscopically, by external or combined approaches. Optimal clinical management requires a long-term follow-up of all cases.

## Competing interests

The authors declare that they have no competing interests.
